# Identifying CDCA4 as a Radiotherapy Resistance-Associated Gene in Colorectal Cancer by an Integrated Bioinformatics Analysis Approach

**DOI:** 10.3390/genes16060696

**Published:** 2025-06-09

**Authors:** Lin Chen, Yawei Gao, Zhiqing Hu, Jingwen Si, Yuchao Zhang, Qingping Cai

**Affiliations:** 1 Department of Gastrointestinal Surgery, Department of General Surgery, Shanghai East Hospital, School of Life Sciences and Technology, Tongji University, Shanghai 200120, China; 13651876639@163.com (L.C.); gaoyawei@tongji.edu.cn (Y.G.); 2 Department of Gastrointestinal Surgery, Department of General Surgery, Shanghai East Hospital, School of Medicine, Tongji University, Shanghai 200120, China; huzhiqing7178@126.com; 3 Shanghai Key Laboratory of Signaling and Disease Research, Frontier Science Center for Stem Cell Research, School of Life Sciences and Technology, Tongji University, Shanghai 200120, China; 4 CAS Key Laboratory of Tissue Microenvironment and Tumor, Shanghai Institute of Nutrition and Health, Chinese Academy of Sciences, Shanghai 201203, China; zhangyuchao@sibs.ac.cn

**Keywords:** colorectal cancer, whole-exome sequencing, neoadjuvant chemoradiotherapy, radiotherapy resistance, CDCA4, B cell infiltration

## Abstract

Background: Colorectal cancer (CRC) remains one of the most prevalent and fatal malignancies globally, with radiotherapy playing a crucial role in the treatment of locally advanced rectal cancer (LARC). However, the efficacy of radiotherapy is limited by significant resistance, with only a small proportion of patients achieving a pathologic complete response (PCR) to neoadjuvant chemoradiotherapy (nCRT). This study aims to uncover the genetic and molecular factors contributing to radiotherapy resistance in CRC through an integrated analysis of germline mutations, transcriptomic data, and immune microenvironment characteristics. Methods: Whole-exome sequencing (WES) was performed on tumor samples from 12 LARC patients. Transcriptomic data from the TCGA-READ and GSE150082 (LARC with chemoradiotherapy) cohorts were integrated with WES findings. The independent cohort GSE190826 (neoadjuvant therapy in rectal cancer) dataset was utilized to validate the WES data. Single-cell RNA sequencing (scRNA-seq) analysis of GSE132465 (primary CRC) resolved cellular heterogeneity. A random forest algorithm was employed to develop a predictive gene signature. Results: Our findings reveal a mutational landscape associated with radiotherapy resistance, identifying specific germline mutations linked to treatment outcomes. Differential gene expression analysis highlighted pathways involved in DNA replication, DNA repair, and immune regulation, with a focus on the tumor immune microenvironment (TIME). A gene signature, including CDCA4, FANCA, PBRM1, RPL13, and C12orf43, was developed to predict radiotherapy response. Notably, CDCA4 expression was significantly associated with tumor mutation burden (TMB) and microsatellite instability (MSI), and it plays a crucial role in regulating B cell infiltration in the tumor microenvironment. Conclusions: Our study provides novel insights into the molecular mechanisms of radiotherapy resistance in CRC and proposes CDCA4 and B cell-related immune features as potential biomarkers for patient stratification and personalized treatment strategies.

## 1. Introduction

According to 2022 global cancer data, colorectal cancer (CRC) represents approximately 10% of the worldwide cancer burden, with 1.9 million diagnoses and 904,000 fatalities. Globally, CRC is the third most commonly diagnosed malignancy and the second leading cause of cancer-related deaths [1]. Radiotherapy is adaptable for targeting various malignancies at different anatomical locations and is a routine treatment used in nearly two-thirds of cancer cases, often serving as a curative alternative to surgery [2]. Radiotherapy optimizes therapeutic efficacy by adjusting dosage schedules and fractionation parameters, effectively targeting malignancies while preserving adjacent healthy structures [3]. In locally advanced CRC, the standard treatment combines radiotherapy with chemotherapy. Despite this multimodal approach, neoadjuvant chemoradiotherapy (nCRT) yields pathologic complete responses (PCR) in merely 9–20% of patients [4], with 20–40% demonstrating limited therapeutic benefit [5]. Consequently, the development of biomarkers to predict PCR represents a critical unmet need in clinical oncology.

Emerging evidence suggests that germline mutations play a crucial role in modulating tumor response to radiotherapy [6]. Studies have identified specific germline variants associated with altered DNA damage repair, oxidative stress response, and apoptosis, which are key mechanisms influencing radiation sensitivity and resistance. For instance, mutations in genes such as TP53, ATM, BRCA1/2, and CHEK2 have been linked to impaired DNA repair capacity, potentially contributing to increased resistance to radiation-induced damage [7,8]. Moreover, genome-wide association studies (GWASs) have identified inherited genetic variants linked to heterogeneous treatment responses in patients undergoing radiotherapy [9]. Building upon this foundation, our study aims to identify germline mutation-associated correlates of radiation resistance by leveraging comprehensive genetic profiling and integrative analysis. By uncovering potential biomarkers, we seek to provide novel insights into patient stratification and personalized radiotherapy strategies.

Tumor progression is governed by both epithelial characteristics and the surrounding tumor microenvironment (TME). Within the TME, the tumor immune microenvironment (TIME) has emerged as a key determinant of cancer development and therapeutic efficacy, garnering substantial research focus [10]. Increasing evidence suggests that characterizing the TIME can identify novel prognostic and predictive biomarkers, potentially guiding first-line treatment algorithms [11]. Key components of the TIME have been systematically assessed and integrated into the ‘Immunoscore’, a composite metric that robustly predicts survival outcomes in CRC [12,13]. Consequently, integrating the TIME holds promise for predicting responses to radiotherapy, as supported by recent studies [14]. However, the preliminary findings require further validation in larger patient cohorts. There is an urgent need to develop immune-related gene signatures closely associated with the TIME to predict the efficacy of CRT.

Herein, we performed whole-exome sequencing (WES) analysis on 12 CRC patient samples, by integrating WES findings with transcriptomic profiles from locally advanced rectal cancer (LARC) derived from GSE150082 and The Cancer Genome Atlas–Rectum adenocarcinoma (TCGA-READ). We also used the single-cell RNA sequencing (scRNA-seq) data of tumors from CRC patients (GSE132465 dataset) to annotate our findings. Overall, this research provides a systematic integrative analysis of radiotherapy resistance in CRC, uncovering novel insights into the molecular characteristics driving this complex process.

## 2. Materials and Methods

### 2.1. Patient Cohort

This study included histologically verified locally advanced CRC cases treated with neoadjuvant capecitabine (combined with 50.4 Gy radiation) between 2017 and 2022, followed by definitive surgical resection within 8–11 weeks. Resected tumor specimens were preserved in formalin solution for downstream analysis. This study was approved by the Ethics Committee of East Hospital Affiliated with Tongji University and was conducted in accordance with the 1964 Helsinki Declaration and its subsequent amendments, or comparable ethical standards. We collected tumor tissue from 12 colon cancer patients treated with radiation therapy at East Hospital, with written informed consent obtained from all patients. The 12 samples were categorized into two groups for WES analysis: postoperative pathological assessment classified six cases as radiotherapy-responsive (exhibiting complete or partial pathological remission) and six as non-responsive (with no pathological downstaging observed). The comprehensive clinical characteristics are detailed in Supplementary Table S1.

### 2.2. WES and Data Processing

We selected the aforementioned 12 samples of CRC for WES. Genomic DNA was extracted from tumor tissues using the DNeasy Kit (Qiagen, Germantown, MD, USA) following the manufacturer’s protocol. Sequencing libraries were generated via the Agilent SureSelect Human All Exon system (Illumina-compatible; Agilent Technologies, Santa Clara, CA, USA) and subjected to paired-end sequencing on the Illumina HiSeq2000 platform. The average sequencing depth of the 12 samples was 73.9, and the average base coverage was 84.34%. The detailed sequencing depth and base coverage data for each sample are listed in Supplementary Table S2.

Raw FASTQ files from next-generation sequencing (NGS) were filtered using fastp (version 0.23.4), and the paired-end reads were aligned to the human reference genome GRCh38 using BWA (version 0.7.17-r1198-dirty) [15]. Aligned BAM files were processed with GATK (version 4.3.0.0) for MarkDuplicates and Base Quality Score Recalibration (BQSR) [16]. Germline mutations, including single nucleotide variant (SNVs) and INDELs, were detected using GATK HaplotypeCaller [17] and annotated with ANNOVAR (version 2020-06-08). In the analysis of SNV calling, we considered the potential impacts of the PCR process. We utilized GATK MarkDuplicates to remove duplicates and filtered for low-frequency mutation sites with allele frequencies less than 0.01 in databases such as ExAC_ALL, ExAC_EAS, ALL.sites.2015_08, EAS.sites.2015_08, gnomAD40_exome_AF, and gnomAD40_exome_AF_eas, in order to minimize the effects of PCR artifacts.

### 2.3. Independent Cohort Data Validation

An independent LARC cohort treated with chemoradiotherapy (GSE190826 dataset) was used to validate the results obtained from the WES data [18]. Specifically, we selected transcriptomic data from 36 non-PCR and 18 PCR samples to extract variant information for the specific CDCA4 region, followed by variant filtering and analysis. RNA-seq data were aligned to the reference genome using STAR software (version 2.7.10a) in Two-Pass Mode to improve the detection of splicing events. The alignment results were indexed with SAMtools (version 1.15.1), and we extracted alignments corresponding to the CDCA4 gene and its 10 kb flanking regions. To reduce false-positive variants, PCR duplicates in the target region’s BAM files were marked and removed using Sambamba (version 0.8.2).

We processed the BAM files with GATK’s SplitNCigarReads to accurately identify splice junctions. Readgroup information was added using GATK’s AddOrReplaceReadGroups to ensure necessary metadata for variant detection. Base quality scores were recalibrated with GATK’s BaseRecalibrator and ApplyBQSR via using known variant databases. Variants were called with GATK HaplotypeCaller, and CDCA4 variant information was extracted using BCFtools (version 1.15.1). We filtered the variants with GATK VariantFiltration to remove low-quality ones, applying the following criteria: - **FS > 30.0**: to filter out likely false positives; and - **QD < 2.0**: to filter out low-quality variants. Finally, we annotated the variants with GATK VariantAnnotator, focusing on PASS variants to identify homozygous mutations.

### 2.4. scRNA-Seq Data Analysis

The CRC scRNA-Seq datasets were downloaded from the GEO database with accession number GSE132465 [19]. Raw BCL files were converted to FASTQ files using CellRanger for alignment and quantification [20]. The R package Seurat (v4.4.0) was used to preprocess the scRNA-seq dataset. The data were directly filtered using the parameters min.cells = 3 and min.features = 200. Following this, quality control was performed on the integrated data, setting the threshold for the proportion of mitochondrial genes detected in each cell to be less than 20%. After quality control, the number of cells was 63,689 and the number of genes was 25,655 [21]. The counts data were normalized using the ‘LogNormalize’ method, and the data were scaled using the ScaleData function. We employed the Seurat RunPCA function to compute the principal components (PCs), followed by batch effect correction using Harmony. The PCA plot after batch effect removal with Harmony was obtained. We selected 1 to 40 principal components for subsequent analyses. The FindNeighbors function was utilized to identify neighboring cells, and the FindClusters function was employed to cluster the cells, setting the resolution to 0.6 to obtain between 0 and 22 cell clusters. Visualization was performed using UMAP [22].

### 2.5. Screening Genes with Differential Expression

The GSE150082 dataset [23] was analyzed to detect genes linked to CRT responsiveness through responder versus non-responder comparisons. Expression variations in rectal cancer-associated genes were further evaluated using TCGA-READ. The limma R package was employed to define DEGs (differentially expressed genes) with thresholds of |log_2_ (fold change)| > 1 and adjusted *p*-value < 0.05. To address multiple testing, significant associations were filtered at an FDR < 0.1 via the Benjamini–Hochberg method.

### 2.6. Functional Enrichment Analysis

Functional enrichment analysis of Gene Ontology (GO) terms and Kyoto Encyclopedia of Genes and Genomes (KEGG) pathways was conducted via the R package clusterProfiler to assess cellular functional states [24]. Terms with a *p*-value < 0.05 were considered significantly enriched using the functions “enrichGO” and “enrichKEGG”. Gene Set Enrichment Analysis (GSEA) was conducted using the “GSEA” function in clusterProfiler, leveraging the MSigDB collections [25]. Immune infiltration scores for the responder and non-responder groups were calculated based on single-sample GSEA (ssGSEA) [26]. Infiltration level variations across groups were statistically assessed via the non-parametric Wilcoxon rank-sum test.

### 2.7. Protein–Protein Interaction Network

The STRING database was utilized to generate a CDCA4-centric PPI network, which was subsequently visualized through Cytoscape (v3.9.1). Topological analysis via the cytoHubba plugin identified the five highest-ranked hub genes within the network.

### 2.8. Genomic Heterogeneity Analysis

The TCGA Pan-Cancer harmonized dataset was retrieved via the UCSC Xena browser (https://xenabrowser.net/, accessed on 4 June 2024), from which CDCA4 expression profiles were systematically extracted. Furthermore, somatic mutation data (MuTect2-processed, Level 4) were acquired to complement genomic analyses [27] for all TCGA cases from the GDC portal (https://portal.gdc.cancer.gov/, accessed on 20 June 2021). We calculated the Tumor Mutation Burden (TMB) for each tumor using the TMB function in the R package maftools (version 2.8.05) [28]. Simultaneously, we acquired microsatellite instability (MSI) scores for each tumor from a previously published study [29].

### 2.9. Statistical Analysis

Statistical analyses and visualization were implemented in R (v3.6.1). Intergroup comparisons utilized the Wilcoxon rank-sum test (two-group) or one-way ANOVA (multi-group). Statistical significance thresholds were defined as follows: * *p* < 0.05, ** *p* < 0.01, *** *p* < 0.001, **** *p* < 0.0001.

## 3. Results

### 3.1. Mutational Landscape of Resistance to Radiotherapy in CRC

This study enrolled 12 patients with locally advanced CRC, stratified into two groups based on pathological evaluation following nCRT: 6 responders (pathologic complete/partial response) and 6 non-responders (no pathologic downstaging). The demographic, treatment, and tumor characteristics are summarized in Supplementary Table S1. The median age of the cohort was 62.5 years, comprising four males and eight females. Histological examination of the tumor samples revealed 11 cases of adenocarcinoma and 1 case of signet-ring cell carcinoma. After neoadjuvant therapy, one patient experienced recurrence resulting in death, while the remaining patients were alive. To investigate the factors associated with radiotherapy resistance, we conducted WES to identify genetic mutations related to radiotherapy.

**Figure 1:**
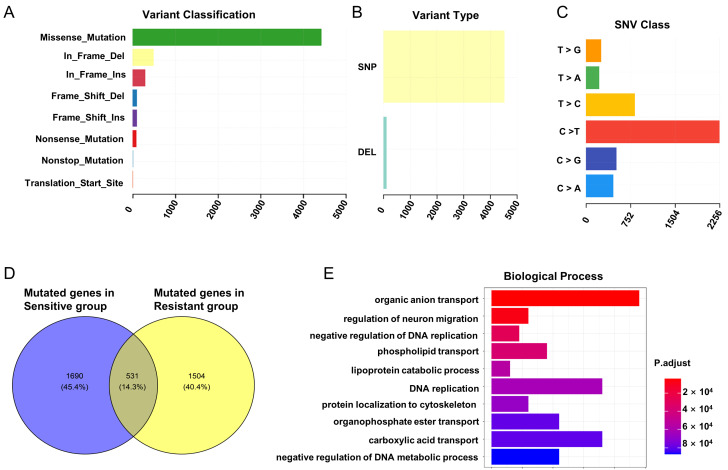
Mutational landscape of resistance to radiotherapy in CRC. (**A**) Variant classification summary in WES. (**B**) Variant type summary in WES. (**C**) SNV class summary in WES. (**D**) Specifically mutated genes in the sensitive and resistant to radiotherapy groups. (**E**) GO biological process function enrichment analysis of these mutated genes associated with CRC radiotherapy resistance.

The global landscape of germline mutations in CRC, generated from the WES data of six radiotherapy-sensitive and six radiotherapy-resistant patients, is shown in Figure 1. A total of 3725 mutated genes and 5512 loci mutations were identified, including 4425 missense mutations (Figure 1A). Among these missense mutations, 4314 were single nucleotide polymorphisms (SNPs) and 111 were deletion mutations (Figure 1B). C>T mutation is the most common SNV, accounting for nearly half (Figure 1C). Additionally, the variant gene profiles across the 12 samples and the variant classification summary are presented in Supplementary Figure S1A,B. The top ten most frequently mutated genes are also listed in the WES data (Supplementary Figure S1C).

Subsequently, to explore the germline mutations that correlate with the radiotherapy resistance, we investigated the exclusive gene mutations in the sensitive or resistant group. In total, 1690 mutated genes in the sensitive group and 1504 mutated genes in the resistant group were identified (Figure 1D). To uncover the biological functions of these mutated genes, we performed GO pathway analysis, and the result demonstrated that those genes were mainly enriched in organic anion transport, DNA replication, and negative regulation of DNA replication (Figure 1E).

### 3.2. Identification and Functional Enrichment of DEGs in Radiation-Resistant Rectal Cancer Cohort

**Figure 2:**
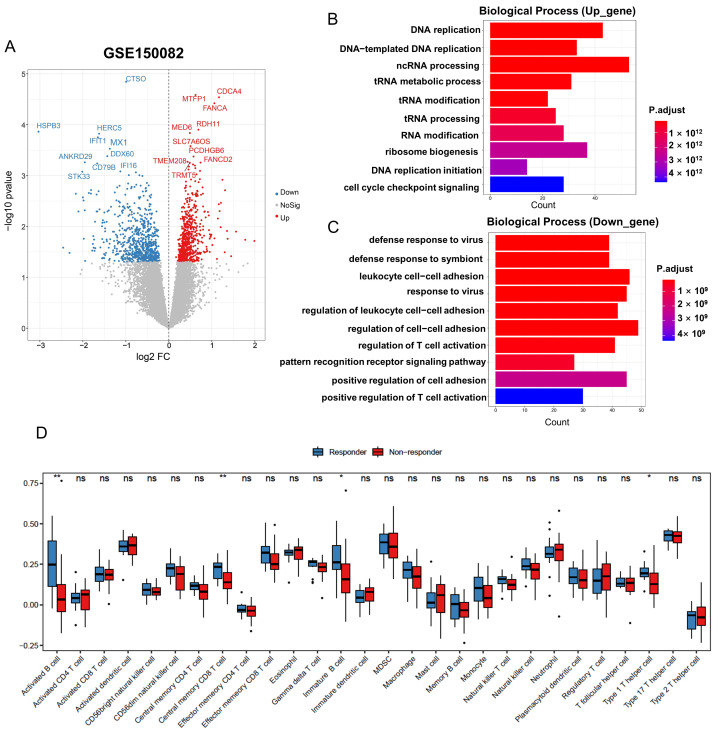
Identification and functional enrichment of DEGs in radiation-resistant rectal cancer cohort. (**A**) Volcano plot of DEGs between responders and non-responders to chemoradiotherapy in LARC patients in GSE150082. (**B**) GO biological process function enrichment analysis of the Up and (**C**) Down genes associated with radiation-resistant rectal cancer. (**D**) Differences in infiltration of 28 immune cells between responder and non-responder groups were calculated using the ssGSEA algorithm. * *p* < 0.05, ** *p* < 0.01.

**Figure 3:**
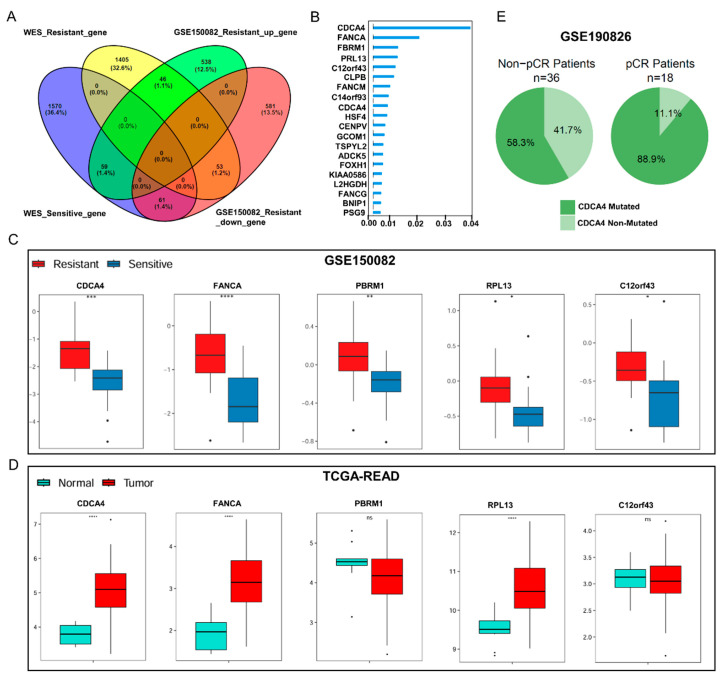
WES combined transcriptome analysis. (**A**) Venn diagram analysis of the upregulated genes in the radiation-resistant group from the GSE150082 and the mutated genes identified in the WES. (**B**) Random forest analysis of the identified mutated genes. (**C**) The expression differences of the top five genes (CDCA4, FANCA, PBRM1, RPL13, and C12orf43) between the resistant and sensitive groups in the GSE150082 dataset. (**D**) Differential expression analysis of the top five genes in the TCGA-READ cohort. (**E**) Mutation frequency of CDCA4 in the non-PCR and PCR groups within the GSE190826 dataset. * *p* < 0.05, ** *p* < 0.01, *** *p* < 0.001, **** *p* < 0.0001.

To narrow down the genes that correlate with radiotherapy resistance, we investigated how many of those mutated genes were also differently expressed between the radiation-resistant and radiation-sensitive groups. We conducted transcriptomic analysis using bulk RNA-seq data from 16 sensitive and 23 resistant patients who received standard long-course pelvic radiotherapy and capecitabine chemotherapy (GSE150082 dataset). A total of 1338 DEGs were identified, with 643 genes upregulated and 695 genes downregulated in the non-responder rectal cancer cohort (Figure 2A, Supplementary Figure S2). Among the radiotherapy-related mutated genes, there were 46 genes in the WES-resistant group and 59 genes in the WES-sensitive group that were also consistent with the different expression between the radiation-resistant and radiation-sensitive groups (Figure 3A).

Functional enrichment analysis revealed that upregulated genes in the radiation-resistant group were enriched in several pathways, such as DNA replication, DNA repair, and cell cycle checkpoint signaling (Figure 2B). In contrast, downregulated genes were enriched in pathways such as response to viruses, regulation of cell–cell adhesion, and regulation of T cell activation (Figure 2C). Additionally, we calculated the enrichment scores for hallmark pathways in tumors and evaluated the differences between the radiation-sensitive and radiation-resistant groups using Gene Set Variation Analysis (GSVA). Significant enrichment and notable differences were observed in several hallmark pathways, including hallmark_apical_surface, hallmark_protein_secretion, and hallmark_pi3k_akt_mtor_signaling (Supplementary Figure S3). Next, we investigated immune cell infiltrates inferred from bulk RNA-seq data. We observed significantly more activated B cells, immature B cells, central memory CD8+ T cells, and type 1 T helper cells in the radiation-sensitive tumor specimens (Figure 2D). The activation of these immune cells may enhance the antitumor immune response within the TME, thereby increasing sensitivity to treatment. In summary, clear shifts in the immune infiltrate composition were observed in the radiation-resistant rectal cancer cohort based on bulk transcriptome analysis.

### 3.3. Development of a Gene Signature for Predicting Radiotherapy Efficacy

To obtain a robust gene signature for predicting responders to radiotherapy, we executed a machine learning method, random forest algorithm, to screen out the optimal gene signature from the identified mutated genes. We identified the top five genes (CDCA4, FANCA, PBRM1, RPL13, and C12orf43) based on *p*-value < 0.01 (Figure 3B). Meanwhile, we compared the expression differences of these genes between the resistant and sensitive groups (Figure 3C). We used receiver operating characteristic (ROC) curves to evaluate the predictive performance of these five genes. All of them had an area under the curve (AUC) greater than 0.7, indicating high accuracy (Supplementary Figure S5). Moreover, differential expression analysis of those five genes in the TCGA-READ cohort confirmed that CDCA4, FANCA, and RPL13 were specifically expressed in the tumor compared to the normal samples (Figure 3D). Due to CDCA4 being identified as the most important factor in the random forest analysis, we selected CDCA4 for further analysis. The WES sequencing results revealed a rare nonsynonymous single nucleotide variant (SNV), rs752564841 (CDCA4 c.G239T, p.R80L), which was identified in exon 2 of the CDCA4 gene (transcripts NM_017955 and NM_145701). This variant leads to an arginine-to-leucine substitution at codon 80. It is extremely rare in the general population, with an allele frequency of less than 0.01 in public databases such as ExAC_ALL and gnomAD v4.0 exome. In silico functional prediction tools suggest a potential deleterious impact: PolyPhen-2 (HDIV) classifies it as possibly damaging (P), while PROVEAN predicts it to be deleterious (D). Subsequently, we validated our findings using the independent LARC cohort (GSE190826 dataset), which investigates the key role of inflammatory fibroblasts in resistance to neoadjuvant therapy. By extracting variant information for CDCA4 from the transcriptomic data of 36 non-PCR and 18 PCR CRC patients prior to chemoradiotherapy, we observed that the mutation frequency of CDCA4 in the PCR group was significantly higher than that in the non-PCR group (Chi-square test, *p* = 0.03) (Figure 3E).

**Figure 4:**
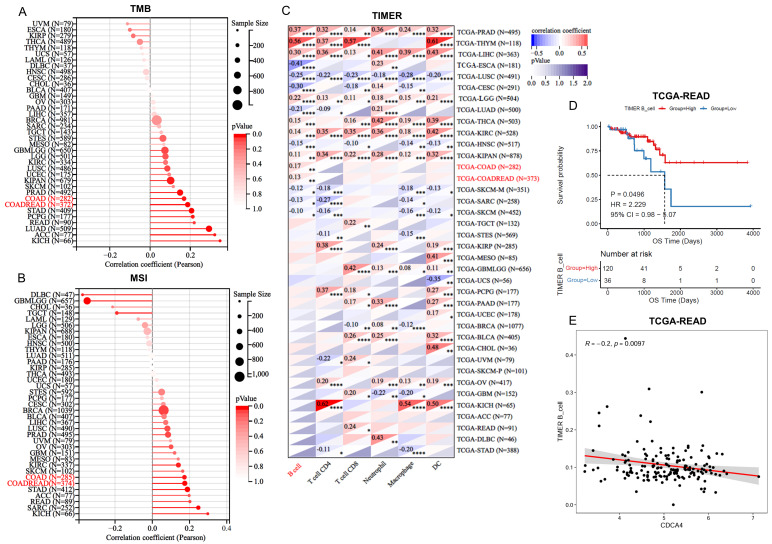
Pan-cancer analysis of CDCA4 in relation to immune infiltration. (**A**) TMB analysis of CDCA4 in different cancer types analyzed using the Sangerbox database. (**B**) MSI analysis of CDCA4 in different cancer types analyzed using the Sangerbox database. (**C**) Immune cell infiltration analysis of CDCA4 in different cancer types analyzed using the Sangerbox database. (**D**) Relationship between B cell infiltration and prognosis in TCGA-READ. (**E**) Relationship between B cell infiltration and CDCA4 expression in TCGA-READ. * *p* < 0.05, ** *p* < 0.01, *** *p* < 0.001, **** *p* < 0.0001.

As the TMB and MSI of CRC are known correlates with radiotherapy efficacy or CRC survival outcomes, we investigated whether the expression of CDCA4 in tumors was associated with the TMB and MSI. We analyzed the TMB and MSI profile of COAD and COADREAD patients in TCGA and correlated them with the corresponding expression of CDCA4 in the tumors. The results indicated that CDCA4 is significantly associated with TMB and MSI (Figure 4A,B). The occurrence of MSI is due to functional defects in the DNA mismatch repair system in tumor tissues, consistent with our previous functional enrichment results.

### 3.4. The Regulatory Network of CDCA4 Affects B Cell Development

Using the TCGA RNA-seq data, we further estimated the immune infiltration profile to explore whether CDCA4 was associated with the TIME. Interestingly, correlation analysis revealed that the expression level of CDCA4 is significantly associated with B cell immune infiltration in COAD and COADREAD (Figure 4C). In addition, analysis of the TCGA-READ cohort revealed improved prognosis associated with B cell infiltration (Figure 4D), while CDCA4 expression demonstrated an inverse correlation with B cell infiltration levels (Figure 4E).

**Figure 5:**
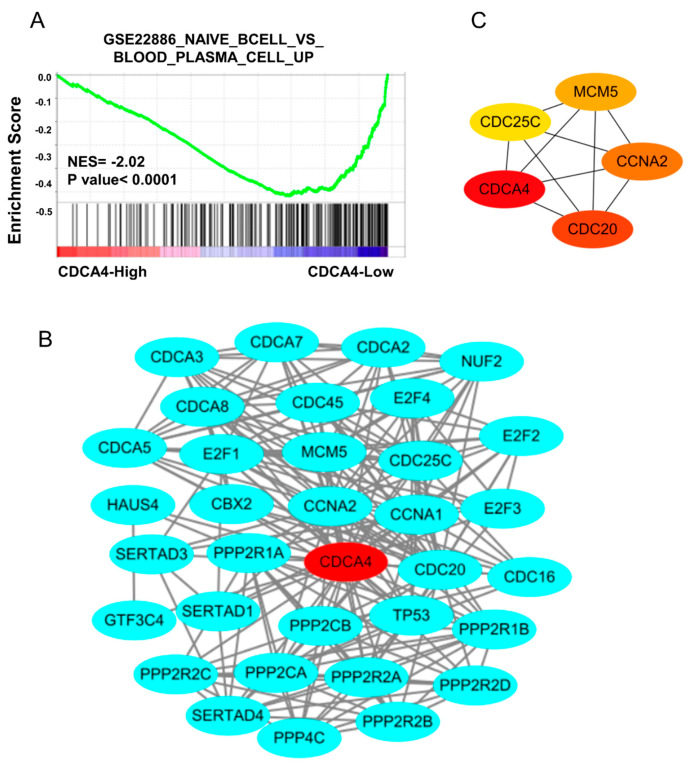
The PPI regulatory network of CDCA4 affects B cell development. (**A**) GSEA of CDCA4 based on the TCGA-READ dataset. (**B**) The PPI network of CDCA4 was constructed using the STRING online database. (**C**) The top five genes of the CDCA4 PPI network were calculated using the MCC algorithm in the cytoHubba plugin of Cytoscape software.

To further investigate the relationship between CDCA4 and B cell infiltration, based on the TCGA-READ datasets, we established the GSEA on the gene sets of “GSE22886_NAIVE_BCELL_VS_BLOOD_PLASMA_CELL_UP” and found that high expression of CDCA4 is negatively correlated with the ratio of naive B cells to mature plasma cells (Figure 5C). Furthermore, based on the STRING database, we constructed a CDCA4 PPI network that was visualized via Cytoscape (Figure 5B). The top four hub genes were screened by the cytoHubba plugin, including CCNA2, CDC20, CDC25C, and MCM5 (Figure 5C). Studies have shown that CCNA2 is involved in the cell cycle and DNA replication processes of B cells [30,31]. CDC20 and CDC25C are highly expressed in diffuse large B cell lymphoma, suggesting that these genes may also influence B cell growth and development [32,33]. In other words, we found that the CDCA4 regulatory network may influence B cell immune infiltration in CRC by regulating the development of B cells.

### 3.5. Single-Cell RNA-Seq Reveals CDCA4 Expression in Epithelial Cells Correlates with Tumor Progression and DNA Repair Pathways

**Figure 6:**
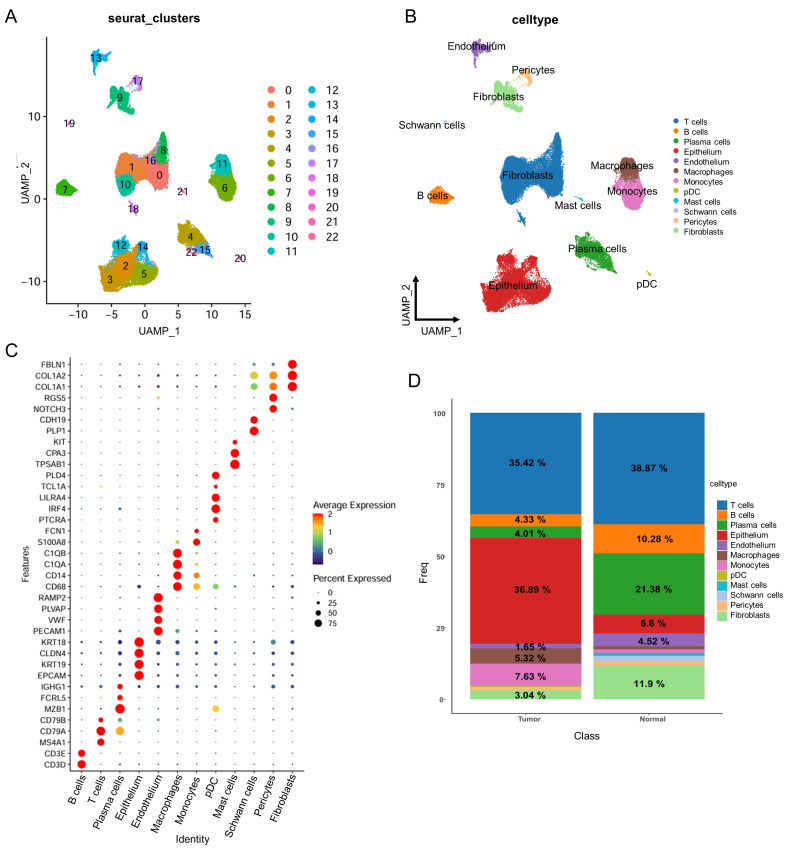
Single-cell RNA-seq analysis in CRC. (**A**) UMAP plots of 22 cell clusterings (n = 63,689 cells) from 23 primary CRC samples and 10 normal mucosal samples by scRNA-seq. (**B**) UMAP plots of 12 types of cells (n = 63,689 cells) from 23 primary CRC samples and 10 normal mucosal samples by scRNA-seq. (**C**) Bubble plot of signature genes for immune cell clusters. Each cell cluster is represented by three specifically expressed genes. (**D**) Bar plot showing the immune cell type proportion in each tumor and normal sample.

To gain a deeper understanding and address the issue of cellular heterogeneity in tumor tissue, we utilized scRNA-seq data from the tumors of CRC patients (GSE132465), which describes the comprehensive cellular landscape and potential cell interactions in CRC. This dataset comprises 23 primary CRC samples and 10 normal mucosal samples. After stringent filtering and quality control, 63,689 cells were retained for further analysis. Using graph-based clustering, these cells were categorized into 22 clusters (Figure 6A). After annotating these cells using marker genes from the CellMarker 2.0 database, the cell clusters were divided into eight subgroups: T cells, B cells, plasma cells, epithelial cells, endothelial cells, macrophages, monocytes, plasmacytoid dendritic cells (pDC), mast cells, Schwann cells, pericytes, and fibroblasts (Figure 6B,C). We observed that the proportion of immune cells, such as B cells and plasma cells, is higher in normal control samples compared to tumor samples. Conversely, epithelial cells are significantly more prevalent in tumor samples (Figure 6D).

**Figure 7:**
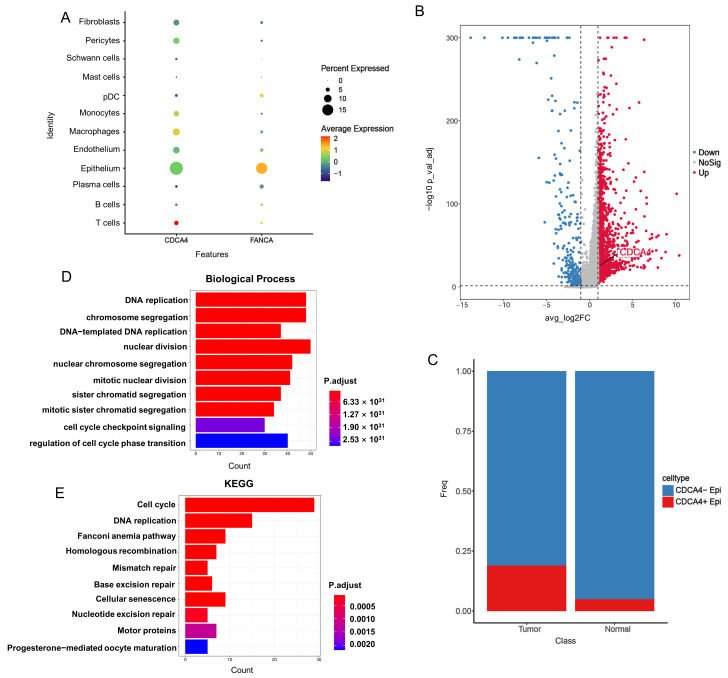
Expression profile of CDCA4 in epithelial cells. (**A**) Bubble plot of CDCA4 and FANCA expression in different cell types. (**B**) Volcano plot of DEGs between the Epithelium-Tumor and Epithelium-Normal groups. (**C**) The proportion of CDCA4+ Epi and CDCA4− Epi in normal and tumor samples. (**D**) GO biological process function enrichment analyses on the upregulated genes in CDCA4+ epithelial cells. (**E**) KEGG pathway analyses on the upregulated genes in CDCA4+ epithelial cells.

Interestingly, we found that CDCA4 and FANCA exhibit higher expression levels in epithelial cells (Figure 7A). We subsequently focused on the differences in epithelial cells and categorized them into the Epithelium-Tumor and Epithelium-Normal groups. This analysis identified 1848 DEGs, among which CDCA4 was upregulated in the Epithelium-Tumor group (Figure 7B). We defined epithelial cells with expression levels of CDCA4 greater than 0 as CDCA4+ Epi and those with no expression as CDCA4− Epi (Figure 7C). By comparing the expression differences between CDCA4+ Epi and CDCA4− Epi cells, we identified 175 DEGs (158 upregulated; 17 downregulated). Finally, we performed GO and KEGG enrichment analyses on the upregulated genes in CDCA4+ epithelial cells. Our functional analysis revealed that the upregulated genes are mainly associated with DNA replication, DNA repair, and the cell cycle (Figure 7D,E). In short, CDCA4+ Epi is closely associated with pathways involved in DNA replication, the cell cycle, and DNA repair. The high expression of genes such as CDCA4 in epithelial cells may be a potential factor contributing to the radiation resistance of CRC.

## 4. Discussion

Patient responses to radiotherapy in CRC vary significantly, with pathological complete response correlating with better outcomes. However, effective methods to identify patients who would benefit from radiotherapy are still lacking. This study investigated the genetic and transcriptomic alterations linked to radiation-resistant CRC. We found that a shared genetic and transcriptomic background contributes to the onset and progression of radiation resistance. Our WES analysis of 12 CRC patients revealed 3725 gene mutations and 5512 loci mutations, with 1504 mutated genes exclusive to the resistant group. GO analysis indicated significant enrichment in pathways related to organic anion transport, DNA replication, and its negative regulation. Transcriptomic data integration showed that genomic variations influence transcriptional profiles. From 1338 DEGs, we identified 46 mutated genes in the resistant group and 59 in the sensitive group. Notably, CDCA4 emerged as a key factor in radiation-resistant CRC through random forest analysis. Additionally, scRNA-seq of 23 primary CRC and 10 normal mucosal samples revealed CDCA4 upregulation in tumor epithelium, with associated genes linked to DNA replication, repair, and the cell cycle, highlighting a common genomic background. Similar research findings indicate that no somatic alterations showed a significant association with responses to neoadjuvant therapy, but KRAS mutations were linked to a faster relapse in patients who undergo consolidation chemotherapy after receiving nCRT. The overexpression of IGF2 and L1CAM was associated with a reduced response to neoadjuvant therapy [34]. Additionally, a study that performed WES on 28 paired tumor samples collected from the same patients who were unresponsive to chemoradiotherapy before and after treatment demonstrated that recurrent mutations in CTDSP2, APC, KRAS, TP53, and NFKBIZ confer a selective advantage to cancer cells, leading to resistance against chemoradiotherapy treatment. Most of these mutated genes were included in our mutation dataset, indicating the accuracy of our sequencing data. Our research presents a novel gene, CDCA4, which is implicated in the radiotherapy resistance process in CRC [35].

Several studies have explored the role of germline mutations in influencing cancer prognosis and response to radiotherapy. Herein, our study identifies additional germline variants including CDCA4 that may contribute to radiotherapy resistance, highlighting novel candidate genes that have not been extensively reported in the previous literature. Notably, while some studies suggest that germline mutations can serve as predictive biomarkers for radiation response, the clinical utility of such markers remains under investigation. Our results provide further evidence supporting the stratification of patients based on germline genetic profiles to optimize radiotherapy outcomes. Future studies integrating larger patient cohorts and functional assays will be essential to validate our findings and further elucidate the molecular mechanisms underlying germline mutation-driven radio-resistance.

Tumor-infiltrating B lymphocytes (TIL-Bs), including B cells and plasma cells, play multifaceted roles in antitumor immunity [36]. Our study observed an increased infiltration of activated B cells, immature B cells, and type 1 helper T cells in radiation-sensitive tumors, suggesting that B cell recruitment may counteract radiotherapy resistance. This aligns with recent single-cell studies showing that CD20⁺ B cell infiltration enhances PD-1 antibody efficacy in CRC [37]. Tumor-infiltrated activated B cells are significantly depleted in CRC and can suppress liver metastasis of CRC [38]. Mechanistically, B cells may modulate radiotherapy outcomes through antigen presentation and cytokine regulation [39,40,41].

Recent studies have shown that the CDCA gene family proteins (CDCA2, CDCA3, CDCA4, CDCA5, CDCA7, CDCA8) may serve as potential prognostic and therapeutic targets for CRC [42]. Specifically, CDCA3 mediates p21-dependent proliferation by regulating E2F1 expression in CRC [43]. CDCA5 promotes CRC progression through the activation of the ERK signaling pathway [44]. Interestingly, CDCA2 not only enhances the proliferation of CRC cells by activating the AKT/CCND1 pathway but also reduces the radiosensitivity of gastric adenocarcinoma by activating the PI3K/AKT pathway [45,46]. Analysis of WES data from 12 patients revealed distinct CDCA family mutation patterns; specifically, radiotherapy-sensitive individuals harbored mutations in CDCA4, CDCA5, and CDCA8, while a CDCA7 mutation was exclusively detected in a radiation-resistant case (Supplementary Figure S7A). Notably, CDCA3, CDCA4, CDCA5, and CDCA8 exhibited elevated expression trends in the radiation-resistant cohorts (Supplementary Figure S7B), consistent with the TCGA-READ database findings showing preferential CDCA family overexpression in tumor specimens (Supplementary Figure S7C). These results suggest that members of the CDCA protein family, particularly CDCA4, CDCA5, and CDCA8, may act as potential mediators of radiotherapy resistance, potentially through mutation-induced expression downregulation.

CDCA4 has been identified as a gene expressed specifically in hematopoietic progenitor cells, not in hematopoietic stem cells, and is thought to play a crucial role in cell cycle regulation [47]. CDCA4 is upregulated in non-small cell lung cancer [48], breast cancer [49], osteosarcoma [50], and ovarian cancer [51], making it a potential biomarker and key mediator in various malignancies. Our study extends its role to CRC, showing its overexpression in radiotherapy-resistant tumors. Through single-cell sequencing analysis, we found this malignant phenotype is primarily due to the high expression of CDCA4 in epithelial cells within tumor tissues. Epithelial cells play multiple critical roles in the TIME [52]; they are not only the origin cells of the tumor but also influence tumor growth, immune evasion, and immune responses through various mechanisms [51]. Tumor epithelial cells can secrete various cytokines, chemokines, and growth factors to modulate the immune microenvironment, such as recruiting immunosuppressive cells [53], promoting macrophage polarization to the M2 phenotype [54], and interacting with stromal cells (e.g., fibroblasts) to reshape the TME [55]. Therefore, we hypothesize that CDCA4+ epithelial cells play a significant role in the TIME and may contribute to radiotherapy resistance in CRC. Targeted elimination of the CDCA4+ epithelial cell population could be a novel approach for CRC therapy.

In general, by integrating WES, transcriptome analysis, and single-cell RNA-seq analysis, we found that radiation-resistant CRC might be associated with the infiltration of immune cells, particularly with a reduction in B cell numbers, in the radiotherapy-resistant group. It is plausible to infer that the composition of the TME has significant implications for CRC treatment. Furthermore, our novel findings indicate that high CDCA4 expression may promote tumorigenesis and the progression of CRC. Additionally, epithelial cells with elevated CDCA4 expression in tumor tissues are associated with radiotherapy resistance in CRC patients, suggesting that CDCA4 could serve as a critical therapeutic target. However, further studies are required to validate the effectiveness of these findings in CRC treatment.

## Data Availability

The authors confirm that the data supporting the findings of this study are available within the article. Any additional information is available from the correspondence author.

## Supplementary Materials

The following supporting information can be downloaded at: https://www.mdpi.com/article/10.3390/genes16060696/s1, Figure S1: Summary of CRC mutations in WES; Figure S2: Heatmap of differentially expressed genes between the responder and non-responder in GSE150082; Figure S3: the hallmark pathways in tumors via GSVA analysis; Figure S4: KEGG pathways in tumors via GSVA analysis; Figure S5: ROC curves to evaluate the predictive performance of these five genes; Figure S6: Presentation of five mutated genes and loci identified in WES; Figure S7: Presentation of CDCA mutated genes and loci identified in WES; Table S1: Clinical information of 12 patients with CRC; Table S2: The depth and base coverage of WES-seq.

## Abbreviations

CRC	Colorectal cancer
LARC	Locally advanced rectal cancer
pCR	Pathologic complete response
nCRT	Neoadjuvant chemoradiotherapy
WES	Whole-exome sequencing
scRNA-seq	Single-cell RNA sequencing
TIME	Tumor immune microenvironment
TMB	Tumor mutation burden
MSI	Microsatellite instability
GWASs	Genome-wide association studies
TME	Tumor microenvironment
TCGA-READ	The Cancer Genome Atlas–Rectum adenocarcinoma
NGS	Next-generation sequencing
SNV	Single nucleotide variant
GO	Gene Ontology
KEGG	Kyoto Encyclopedia of Genes and Genomes
GSEA	Gene Set Enrichment Analysis
ssGSEA	Single-sample GSEA
SNPs	Single nucleotide polymorphisms
GSVA	Gene Set Variation Analysis
ROC	Receiver operating characteristic
AUC	Area under the curve
TIL-Bs	Tumor-infiltrating B lymphocytes

## References

[1] Bray F., Laversanne M., Sung H., Ferlay J., Siegel R.L., Soerjomataram I., Jemal A. (2024). Global cancer statistics 2022: GLOBOCAN estimates of incidence and mortality worldwide for 36 cancers in 185 countries. CA Cancer J. Clin..

[2] Kamran S.C., Lennerz J.K., Margolis C.A., Liu D., Reardon B., Wankowicz S.A., Van Seventer E.E., Tracy A., Wo J.Y., Carter S.L. (2019). Integrative Molecular Characterization of Resistance to Neoadjuvant Chemoradiation in Rectal Cancer. Clin. Cancer Res..

[3] Brown J.M., Carlson D.J., Brenner D.J. (2014). The tumor radiobiology of SRS and SBRT: Are more than the 5 Rs involved?. Int. J. Radiat. Oncol. Biol. Phys..

[4] Roh M.S., Colangelo L.H., O’Connell M.J., Yothers G., Deutsch M., Allegra C.J., Kahlenberg M.S., Baez-Diaz L., Ursiny C.S., Petrelli N.J. (2009). Preoperative multimodality therapy improves disease-free survival in patients with carcinoma of the rectum: NSABP R-03. J. Clin. Oncol..

[5] Minsky B.D., Cohen A.M., Kemeny N., Enker W.E., Kelsen D.P., Reichman B., Saltz L., Sigurdson E.R., Frankel J. (1992). Enhancement of radiation-induced downstaging of rectal cancer by fluorouracil and high-dose leucovorin chemotherapy. J. Clin. Oncol..

[6] Thariat J., Chevalier F., Orbach D., Ollivier L., Marcy P.Y., Corradini N., Beddok A., Foray N., Bougeard G. (2021). Avoidance or adaptation of radiotherapy in patients with cancer with Li-Fraumeni and heritable TP53-related cancer syndromes. Lancet Oncol..

[7] Reiner A.S., Watt G.P., Malone K.E., Lynch C.F., John E.M., Knight J.A., Woods M., Liang X., Tischkowitz M., Conti D.V. (2024). Breast Cancer Susceptibility Gene Sequence Variations and Development of Contralateral Breast Cancer. JAMA Netw. Open.

[8] Petry V., Bonadio R.C., Moutinho K., Leite L.S., Testa L., Cohn D., Cagnacci A.C., Kim V.E.H., Del Pilar Estevez-Diz M., Fragoso M. (2024). Frequency of Radiation Therapy-Induced Malignancies in Patients With Li-Fraumeni Syndrome and Early-Stage Breast Cancer and the Influence of Radiation Therapy Technique. Int. J. Radiat. Oncol. Biol. Phys..

[9] Namba S., Saito Y., Kogure Y., Masuda T., Bondy M.L., Gharahkhani P., Gockel I., Heider D., Hillmer A., Jankowski J. (2023). Common Germline Risk Variants Impact Somatic Alterations and Clinical Features across Cancers. Cancer Res..

[10] Qian L., Lai X., Gu B., Sun X. (2022). An Immune-Related Gene Signature for Predicting Neoadjuvant Chemoradiotherapy Efficacy in Rectal Carcinoma. Front. Immunol..

[11] Taube J.M., Galon J., Sholl L.M., Rodig S.J., Cottrell T.R., Giraldo N.A., Baras A.S., Patel S.S., Anders R.A., Rimm D.L. (2018). Implications of the tumor immune microenvironment for staging and therapeutics. Mod. Pathol..

[12] Mlecnik B., Bindea G., Angell H.K., Maby P., Angelova M., Tougeron D., Church S.E., Lafontaine L., Fischer M., Fredriksen T. (2016). Integrative Analyses of Colorectal Cancer Show Immunoscore Is a Stronger Predictor of Patient Survival Than Microsatellite Instability. Immunity.

[13] Galon J., Costes A., Sanchez-Cabo F., Kirilovsky A., Mlecnik B., Lagorce-Pages C., Tosolini M., Camus M., Berger A., Wind P. (2006). Type, density, and location of immune cells within human colorectal tumors predict clinical outcome. Science.

[14] Lynch C., Pitroda S.P., Weichselbaum R.R. (2024). Radiotherapy, immunity, and immune checkpoint inhibitors. Lancet Oncol..

[15] Alganmi N., Abusamra H. (2023). Evaluation of an optimized germline exomes pipeline using BWA-MEM2 and Dragen-GATK tools. PLoS ONE.

[16] Hintzsche J.D., Robinson W.A., Tan A.C. (2016). A Survey of Computational Tools to Analyze and Interpret Whole Exome Sequencing Data. Int. J. Genom..

[17] Lin Y.L., Chang P.C., Hsu C., Hung M.Z., Chien Y.H., Hwu W.L., Lai F., Lee N.C. (2022). Comparison of GATK and DeepVariant by trio sequencing. Sci. Rep..

[18] Nicolas A.M., Pesic M., Engel E., Ziegler P.K., Diefenhardt M., Kennel K.B., Buettner F., Conche C., Petrocelli V., Elwakeel E. (2022). Inflammatory fibroblasts mediate resistance to neoadjuvant therapy in rectal cancer. Cancer Cell.

[19] Lee H.O., Hong Y., Etlioglu H.E., Cho Y.B., Pomella V., Van den Bosch B., Vanhecke J., Verbandt S., Hong H., Min J.W. (2020). Lineage-dependent gene expression programs influence the immune landscape of colorectal cancer. Nat. Genet..

[20] Zheng G.X., Terry J.M., Belgrader P., Ryvkin P., Bent Z.W., Wilson R., Ziraldo S.B., Wheeler T.D., McDermott G.P., Zhu J. (2017). Massively parallel digital transcriptional profiling of single cells. Nat. Commun..

[21] Stuart T., Butler A., Hoffman P., Hafemeister C., Papalexi E., Mauck W.M., Hao Y., Stoeckius M., Smibert P., Satija R. (2019). Comprehensive Integration of Single-Cell Data. Cell.

[22] Dann E., Henderson N.C., Teichmann S.A., Morgan M.D., Marioni J.C. (2022). Differential abundance testing on single-cell data using k-nearest neighbor graphs. Nat. Biotechnol..

[23] Sendoya J.M., Iseas S., Coraglio M., Golubicki M., Robbio J., Salanova R., Kujaruk M., Mikolaitis V., Rizzolo M., Ruiz G. (2020). Pre-Existing Tumoral B Cell Infiltration and Impaired Genome Maintenance Correlate with Response to Chemoradiotherapy in Locally Advanced Rectal Cancer. Cancers.

[24] Wu T., Hu E., Xu S., Chen M., Guo P., Dai Z., Feng T., Zhou L., Tang W., Zhan L. (2021). clusterProfiler 4.0: A universal enrichment tool for interpreting omics data. Innovation.

[25] Liberzon A., Birger C., Thorvaldsdottir H., Ghandi M., Mesirov J.P., Tamayo P. (2015). The Molecular Signatures Database (MSigDB) hallmark gene set collection. Cell Syst..

[26] Peng Z., Ye M., Ding H., Feng Z., Hu K. (2022). Spatial transcriptomics atlas reveals the crosstalk between cancer-associated fibroblasts and tumor microenvironment components in colorectal cancer. J. Transl. Med..

[27] Beroukhim R., Mermel C.H., Porter D., Wei G., Raychaudhuri S., Donovan J., Barretina J., Boehm J.S., Dobson J., Urashima M. (2010). The landscape of somatic copy-number alteration across human cancers. Nature.

[28] Mayakonda A., Lin D.C., Assenov Y., Plass C., Koeffler H.P. (2018). Maftools: Efficient and comprehensive analysis of somatic variants in cancer. Genome Res..

[29] Bonneville R., Krook M.A., Kautto E.A., Miya J., Wing M.R., Chen H.Z., Reeser J.W., Yu L., Roychowdhury S. (2017). Landscape of Microsatellite Instability Across 39 Cancer Types. JCO Precis. Oncol..

[30] Loo S.K., Ab Hamid S.S., Musa M., Wong K.K. (2018). DNMT1 is associated with cell cycle and DNA replication gene sets in diffuse large B-cell lymphoma. Pathol. Res. Pract..

[31] Maguire A., Chen X., Wisner L., Malasi S., Ramsower C., Kendrick S., Barrett M.T., Glinsmann-Gibson B., McGrath M., Rimsza L.M. (2019). Enhanced DNA repair and genomic stability identify a novel HIV-related diffuse large B-cell lymphoma signature. Int. J. Cancer.

[32] Hu K., Li B., Ma R., Yi H., Xu Z., Peng Y., Yu D., Wu H., Cheng T., Lu Y. (2021). Anti-DLBCL efficacy of DCZ0825 in vitro and in vivo: Involvement of the PI3K-AKT-mTOR/JNK pathway. Acta Biochim. Biophys. Sin..

[33] Sun C., Li M., Feng Y., Sun F., Zhang L., Xu Y., Lu S., Zhu J., Huang J., Wang J. (2020). MDM2-P53 Signaling Pathway-Mediated Upregulation of CDC20 Promotes Progression of Human Diffuse Large B-Cell Lymphoma. OncoTargets Ther..

[34] Chatila W.K., Kim J.K., Walch H., Marco M.R., Chen C.T., Wu F., Omer D.M., Khalil D.N., Ganesh K., Qu X. (2022). Genomic and transcriptomic determinants of response to neoadjuvant therapy in rectal cancer. Nat. Med..

[35] Yang J., Lin Y., Huang Y., Jin J., Zou S., Zhang X., Li H., Feng T., Chen J., Zuo Z. (2019). Genome landscapes of rectal cancer before and after preoperative chemoradiotherapy. Theranostics.

[36] Laumont C.M., Banville A.C., Gilardi M., Hollern D.P., Nelson B.H. (2022). Tumour-infiltrating B cells: Immunological mechanisms, clinical impact and therapeutic opportunities. Nat. Rev. Cancer.

[37] Ji L., Fu G., Huang M., Kao X., Zhu J., Dai Z., Chen Y., Li H., Zhou J., Chu X. (2024). scRNA-seq of colorectal cancer shows regional immune atlas with the function of CD20(+) B cells. Cancer Lett..

[38] Xu Y., Wei Z., Feng M., Zhu D., Mei S., Wu Z., Feng Q., Chang W., Ji M., Liu C. (2022). Tumor-infiltrated activated B cells suppress liver metastasis of colorectal cancers. Cell Rep..

[39] Martin A.L., Powell C., Nagy M.Z., Innamarato P., Powers J., Nichols D., Anadon C.M., Chaurio R.A., Kim S., Wang M.H. (2023). Anti-4-1BB immunotherapy enhances systemic immune effects of radiotherapy to induce B and T cell-dependent anti-tumor immune activation and improve tumor control at unirradiated sites. Cancer Immunol. Immunother..

[40] Cho H., Kim J.E., Hong Y.S., Kim S.Y., Kim J., Ryu Y.M., Kim S.Y., Kim T.W. (2022). Comprehensive evaluation of the tumor immune microenvironment and its dynamic changes in patients with locally advanced rectal cancer treated with preoperative chemoradiotherapy: From the phase II ADORE study. Oncoimmunology.

[41] Goff P.H., Riolobos L., LaFleur B.J., Spraker M.B., Seo Y.D., Smythe K.S., Campbell J.S., Pierce R.H., Zhang Y., He Q. (2022). Neoadjuvant Therapy Induces a Potent Immune Response to Sarcoma, Dominated by Myeloid and B Cells. Clin. Cancer Res..

[42] Zhao Z., Feng X., Chen B., Wu Y., Wang X., Tang Z., Huang M., Guo X. (2025). CDCA genes as prognostic and therapeutic targets in Colon adenocarcinoma. Hereditas.

[43] Qian W., Zhang Z., Peng W., Li J., Gu Q., Ji D., Wang Q., Zhang Y., Ji B., Wang S. (2018). CDCA3 mediates p21-dependent proliferation by regulating E2F1 expression in colorectal cancer. Int. J. Oncol..

[44] Shen A., Liu L., Chen H., Qi F., Huang Y., Lin J., Sferra T.J., Sankararaman S., Wei L., Chu J. (2019). Cell division cycle associated 5 promotes colorectal cancer progression by activating the ERK signaling pathway. Oncogenesis.

[45] Gao J., Wang H., Qiu X., Tang J. (2023). E2F3/CDCA2 reduces radiosensitivity in gastric adenocarcinoma by activating PI3K/AKT pathway. Br. J. Radiol..

[46] Feng Y., Qian W., Zhang Y., Peng W., Li J., Gu Q., Ji D., Zhang Z., Wang Q., Zhang D. (2019). CDCA2 promotes the proliferation of colorectal cancer cells by activating the AKT/CCND1 pathway in vitro and in vivo. BMC Cancer.

[47] Fang H., Sheng S., Chen B., Wang J., Mao D., Han Y., Liu Y., Wang X., Gui S., Zhang T. (2022). A Pan-Cancer Analysis of the Oncogenic Role of Cell Division Cycle-Associated Protein 4 (CDCA4) in Human Tumors. Front. Immunol..

[48] Xu C., Cao H., Sui Y., Zhang H., Shi C., Wu J., Ma R., Feng J. (2021). CDCA4 suppresses epithelial-mesenchymal transtion (EMT) and metastasis in Non-small cell lung cancer through modulating autophagy. Cancer Cell Int..

[49] Xu Y., Wu X., Li F., Huang D., Zhu W. (2018). CDCA4, a downstream gene of the Nrf2 signaling pathway, regulates cell proliferation and apoptosis in the MCF-7/ADM human breast cancer cell line. Mol. Med. Rep..

[50] Li J., Zhang F., Li H., Peng F., Wang Z., Peng H., He J., Li Y., He L., Wei L. (2020). Circ_0010220-mediated miR-503-5p/CDCA4 axis contributes to osteosarcoma progression tumorigenesis. Gene.

[51] Gentilini A., Pastore M., Marra F., Raggi C. (2018). The Role of Stroma in Cholangiocarcinoma: The Intriguing Interplay between Fibroblastic Component, Immune Cell Subsets and Tumor Epithelium. Int. J. Mol. Sci..

[52] Hosein A.N., Brekken R.A., Maitra A. (2020). Pancreatic cancer stroma: An update on therapeutic targeting strategies. Nat. Rev. Gastroenterol. Hepatol..

[53] Ke Z., Wang C., Wu T., Wang W., Yang Y., Dai Y. (2020). PAR2 deficiency enhances myeloid cell-mediated immunosuppression and promotes colitis-associated tumorigenesis. Cancer Lett..

[54] Li J., Wei T., Ma K., Zhang J., Lu J., Zhao J., Huang J., Zeng T., Xie Y., Liang Y. (2024). Single-cell RNA sequencing highlights epithelial and microenvironmental heterogeneity in malignant progression of pancreatic ductal adenocarcinoma. Cancer Lett..

[55] Shi K., Li Y., Yang L., Zhang Z., Guo D., Zhang J., Lu Y. (2022). Profiling transcriptional heterogeneity of epithelium, fibroblasts, and immune cells in esophageal squamous cell carcinoma by single-cell RNA sequencing. FASEB J..

