# Supplementary material for: Identifying CDCA4 as a Radiotherapy Resistance-Associated Gene in Colorectal Cancer by an Integrated Bioinformatics Analysis Approach
Source: Genes (Basel). 2025 Jun 9;16(6):696. doi: 10.3390/genes16060696 (PMC12193651; doi:10.3390/genes16060696)
Supplement: Supplementary file 1 [file genes-16-00696-s001.zip › Supplementary figures.pdf]

Supplementary figures

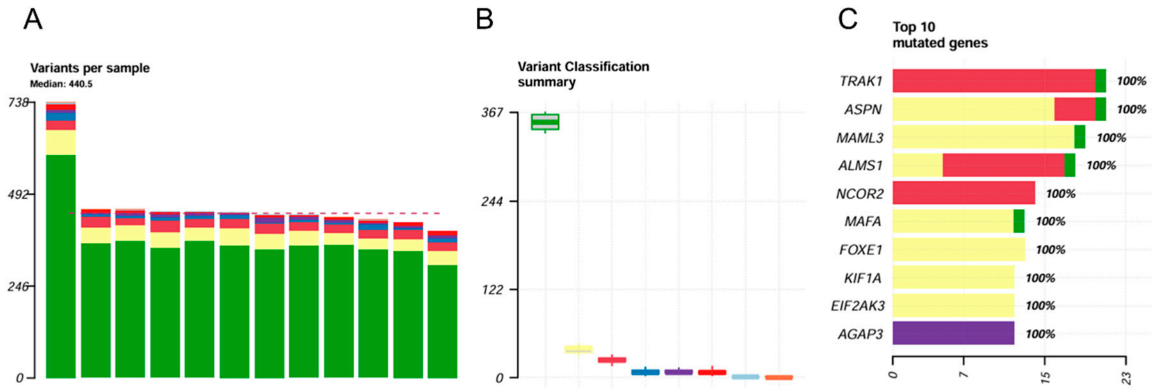

Figure S1. Summary of CRC mutations in WES. (A) the variants gene profiles across the 12 samples and (B) variants classification summary. (C) The top ten most frequently mutated genes.

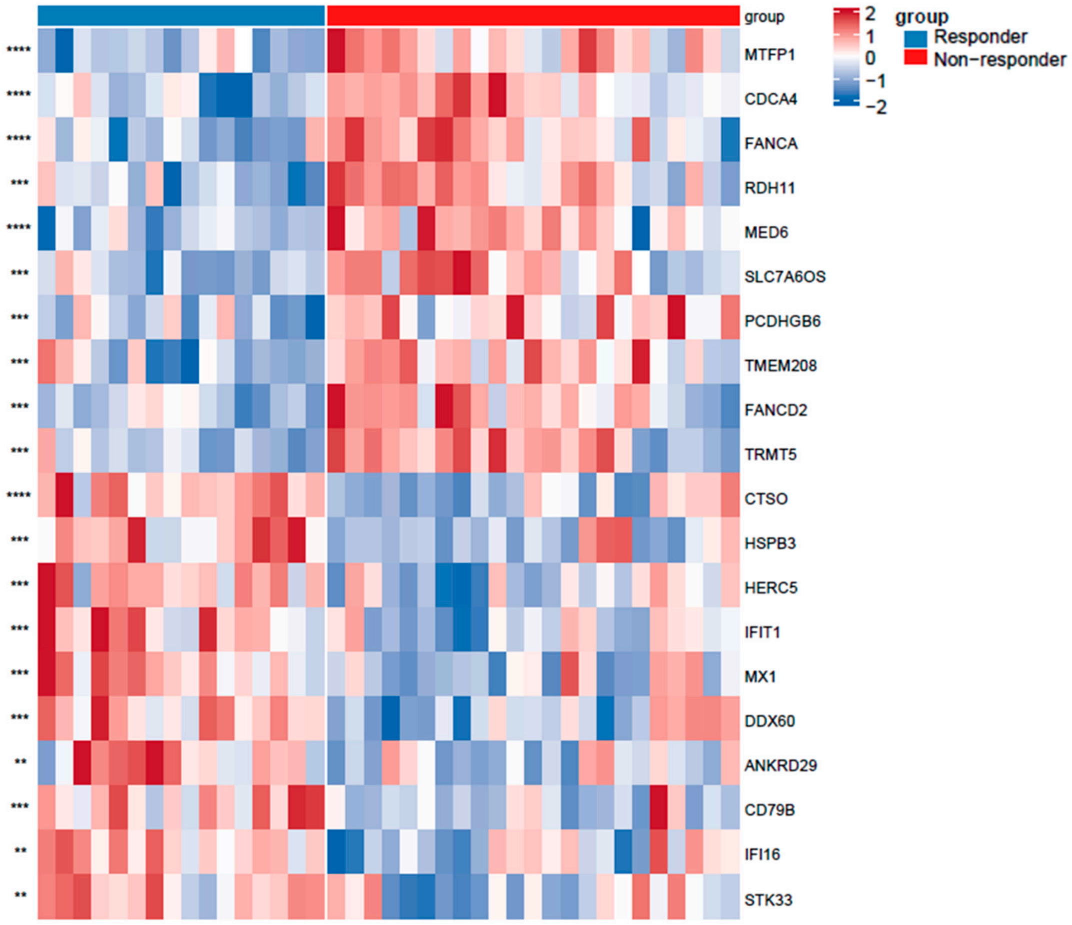

Figure S2. Heatmap of differentially expressed genes between the responder and non-responder in GSE150082.

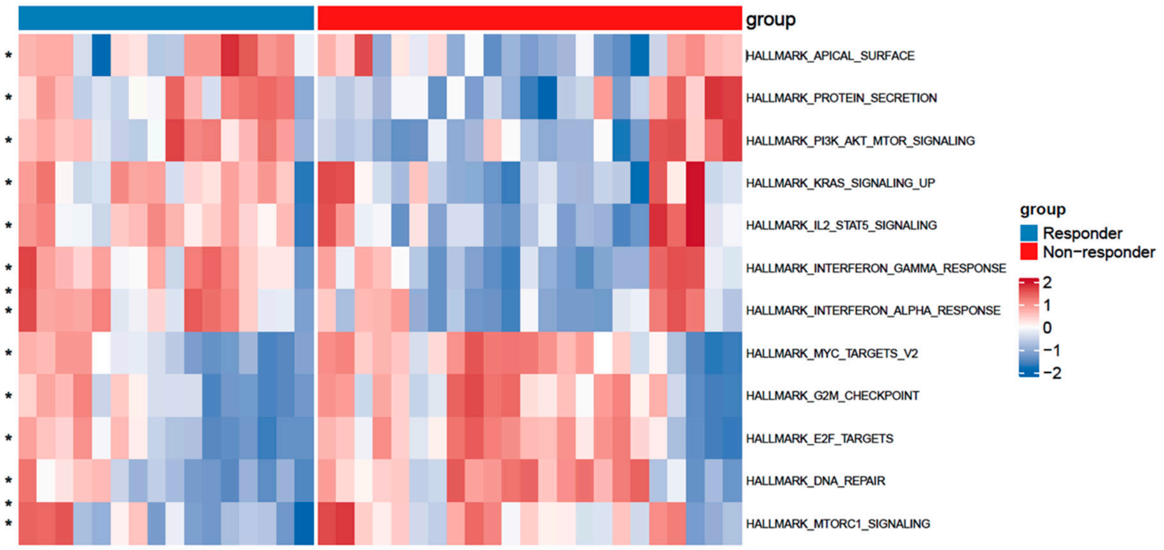

Figure S3. the hallmark pathways in tumors via GSVA analysis.

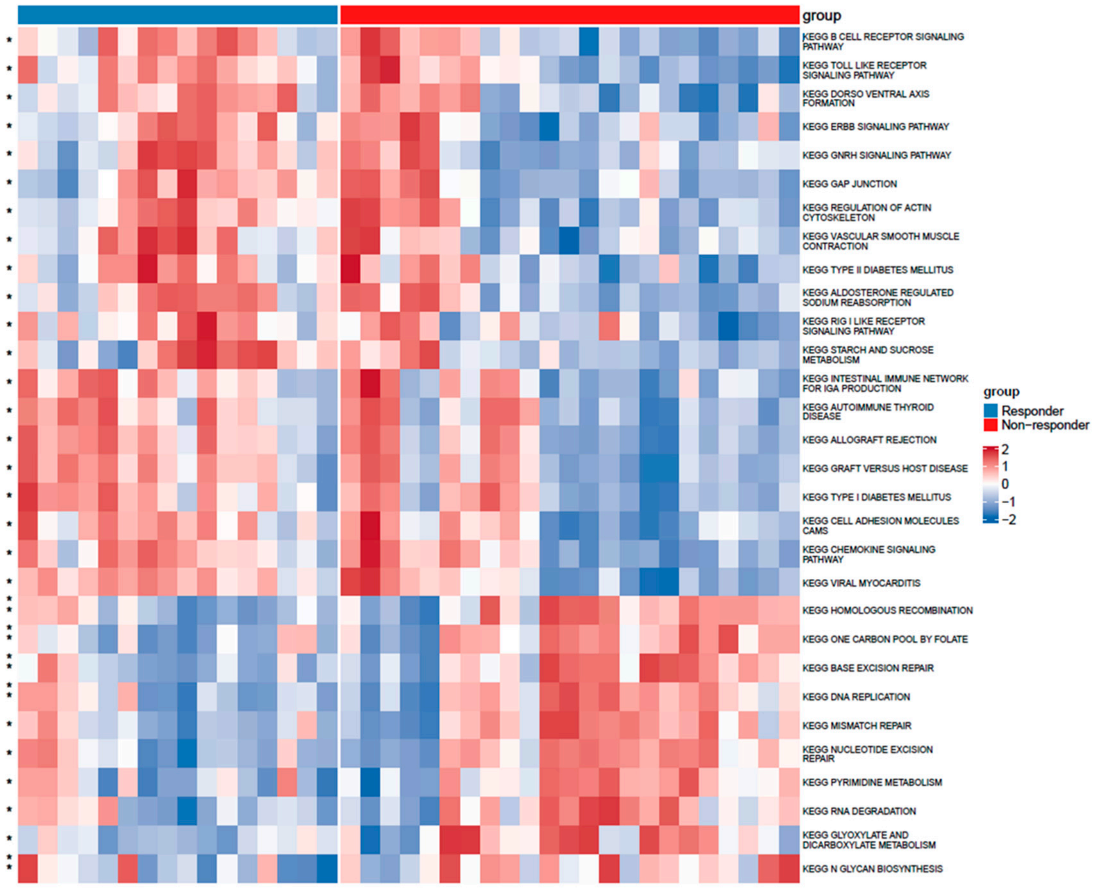

Figure S4. KEGG pathways in tumors via GSVA analysis.

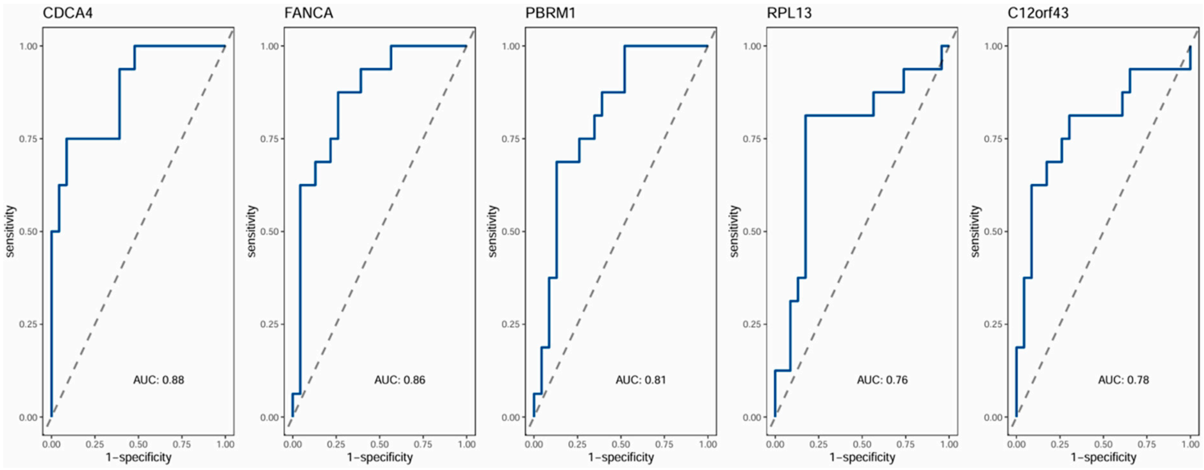

Figure S5. ROC curves to evaluate the predictive performance of these five genes.

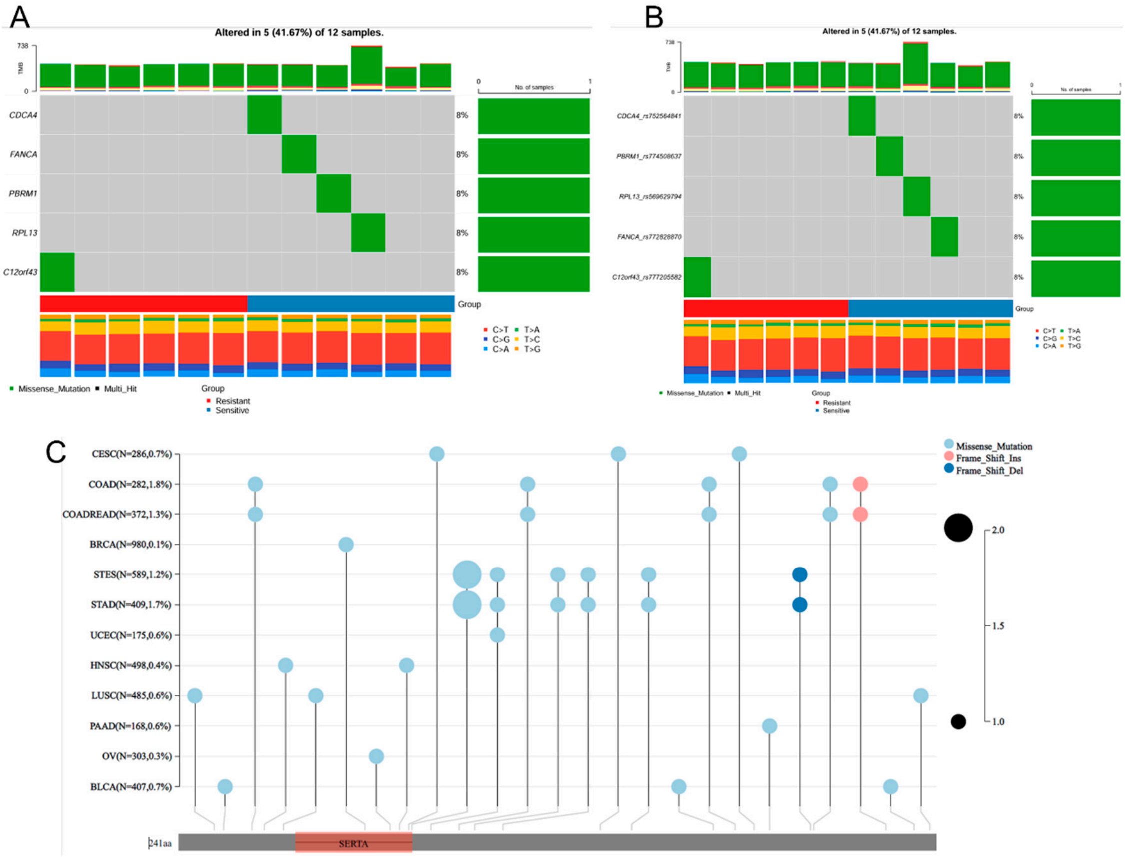

Figure S6. Presentation of five mutated genes and loci identified in WES. (A) Presentation of five mutated genes and (B) loci identified in WES. (C) CDCA4 mutations in pan-cancer studies.

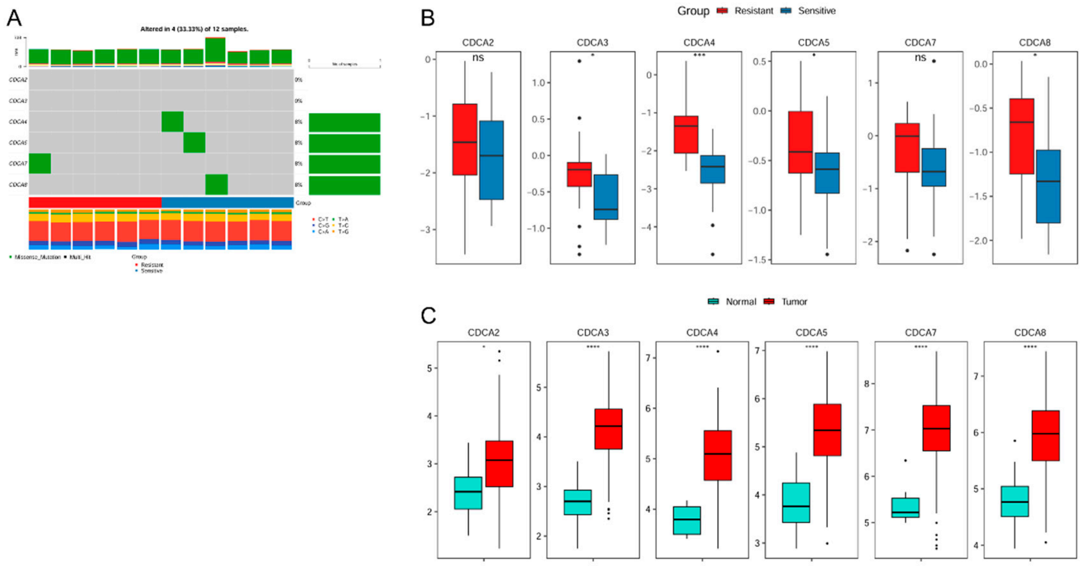

Figure S7. Presentation of CDCA mutated genes and loci identified in WES. (A) Presentation of CDCA mutated genes. (B) Expression levels of CDCA gene family in radiotherapy resistance and sensitive group. (C) Expression levels of CDCA gene family in TCGA-READ.
